# Serum decorin and biglycan levels as predictive biomarkers for lung fibrosis severity and mortality risk in COVID-19 patients

**DOI:** 10.3389/fmed.2024.1463433

**Published:** 2025-01-21

**Authors:** Shaaf Ahmad, Kaleem Maqsood, Farwa Liaqat, Nabila Roohi

**Affiliations:** ^1^King Edward Medical University/Mayo Hospital, Lahore, Punjab, Pakistan; ^2^Institute of Zoology, University of the Punjab, Lahore, Punjab, Pakistan

**Keywords:** COVID-19, decorin, biglycan, proteoglycan, lung fibrosis

## Abstract

**Introduction:**

Individuals experiencing severe symptoms of COVID-19 are at the greatest risk of developing post-COVID lung fibrosis, which significantly impacts long-term health outcomes. This study aims to investigate the predictive potential of serum biomarkers, specifically decorin and biglycan, in assessing the severity and mortality risk among COVID-19 patients.

**Methods:**

For this study, healthy controls and COVID-19 patients (*n* = 240) among them 186 with moderate and 54 with severe symptoms from Ittefaq Hospital and Mayo Hospital, Lahore, Pakistan were recruited satisfying the inclusion and exclusion criteria. Patients were followed up for 2 months. Serum level of decorin and biglycan was evaluated by ELISA. One-way ANOVA and Independent sample “*t*”-test were applied at significance level *p* < 0.05 by using GraphPad Prism.

**Results:**

Decorin levels significantly decreased from controls (43.36 ± 1.14 ng/mL) to moderate (40.24 ± 0.64 ng/mL) and severe COVID-19 patients (35.49 ± 1.00 ng/mL) (*p* = 0.0059). Biglycan levels increased from controls (66.15 ± 2.22 pg/mL) to moderate (70.02 ± 1.57 pg/mL) and severe patients (75.88 ± 1.97 pg/mL) (*p* = 0.0042). In follow-up, survivors had higher decorin (39.6 ± 0.59 ng/mL) than non-survivors (35.84 ± 1.61 ng/mL) (*p* = 0.0319). Biglycan levels were similar between survivors (70.98 ± 1.41 pg/mL) and non-survivors (73.99 ± 3.24 pg/mL) (*p* = 0.459). Higher decorin levels correlate with survival in COVID-19 patients.

**Conclusion:**

Serum decorin and biglycan levels are valuable biomarkers for predicting severity and mortality in COVID-19 patients. Lower decorin and higher biglycan levels correlate with increased disease severity, emphasizing their potential to identify patients at risk for lung fibrosis and guide clinical management.

## 1 Introduction

The COVID-19 pandemic has affected over 776 million people and almost 7.1 million mortalities were reported worldwide (1). The developing clinical symptoms of this infection include cough, headache, difficulty in breathing, sore throat, myalgia, and fever (2). It was observed that after COVID-19 infection, more than 30 per cent of individuals still experience impaired diffusing capacity of the lungs for carbon dioxide (DLCO) and constant lung damage, with 1/3^rd^ of patients exhibiting severe DLCO impairment and lung fibrosis. These respiratory complications can lead to significant morbidity and even mortality may also occur as the lung fibrosis progresses (3). Considering these results, the impact of pulmonary fibrosis following COVID-19 recovery could be significant. Even though the virus is eliminated in patients who have recovered from the coronavirus, the elimination of the cause of pulmonary damage alone does not prevent the development of escalating, fibrotic, irreparable interstitial lung disease (4).

Pulmonary fibrosis may arise either as a result of chronic inflammation or as a primary fibroproliferative process influenced by genetics and age, as seen in idiopathic pulmonary fibrosis (5).

The decorin molecule is a member of the leucine-rich proteoglycan group and plays a crucial role in the assemblage of the extracellular matrix (ECM) and regulates various cellular processes including growth, inflammation, proliferation, cellular adhesion, and fibrogenesis (6). Similarly, biglycan, possessing two glycosaminoglycan chains, is closely localized around the cells. Studies have shown that decorin and biglycan can interact with Transforming Growth Factor Beta (TGF-*β*) *in vitro*, a pivotal pro-fibrotic mediator in tissue fibrosis (7). Due to the localization of biglycan in the pericellular ECM, it likely plays a role in trapping TGF-*β* near cell-surface receptors, thereby facilitating ECM assembly. Whereas decorin is predominantly found in the interstitial ECM (8).

The novel coronavirus SARS-CoV-2 exhibits a great transmission rate, high intensity, and more virulence as compared to previously identified coronaviruses. It mainly targets the respiratory system by triggering a cytokine storm, resulting in extensive inflammation and lung fibrosis. It’s noteworthy that decorin exhibits strong anti-inflammatory properties, inhibits cytokine activity, and prevents fibrillogenesis. These characteristics make it a promising candidate for drug development to address complications associated with COVID-19, particularly in the context of pulmonary fibrosis.

Lung fibrosis presents significant diagnostic challenges, especially in patients recovering from severe COVID-19. Accurate diagnosis is crucial, with high-resolution computed tomography (HRCT) serving as the gold standard, complemented by pulmonary function tests (PFTs) and, when necessary, bronchoscopy with biopsy for definitive confirmation.

Liquid biopsy is emerging as a vital tool for early detection and monitoring treatment response of Lung cancer. Advances in this field highlight the potential of DNA/RNA-based biomarkers, proteins, autoantibodies, and circulating tumor cells (CTCs). Despite promising results, challenges remain in translating these biomarkers into clinical practice, including reliability and specificity (9).

The fundamental goal of this investigation is to determine the level of biglycan and decorin in COVID-19 patients (with moderate and severe symptoms) as compared to the control group. Moreover, the findings of the investigation would help us to anticipate the future risks of lung fibrosis manifested in COVID-19 exposure and other immunosuppressant respiratory diseases.

## 2 Materials and methods

**Figure 1:**
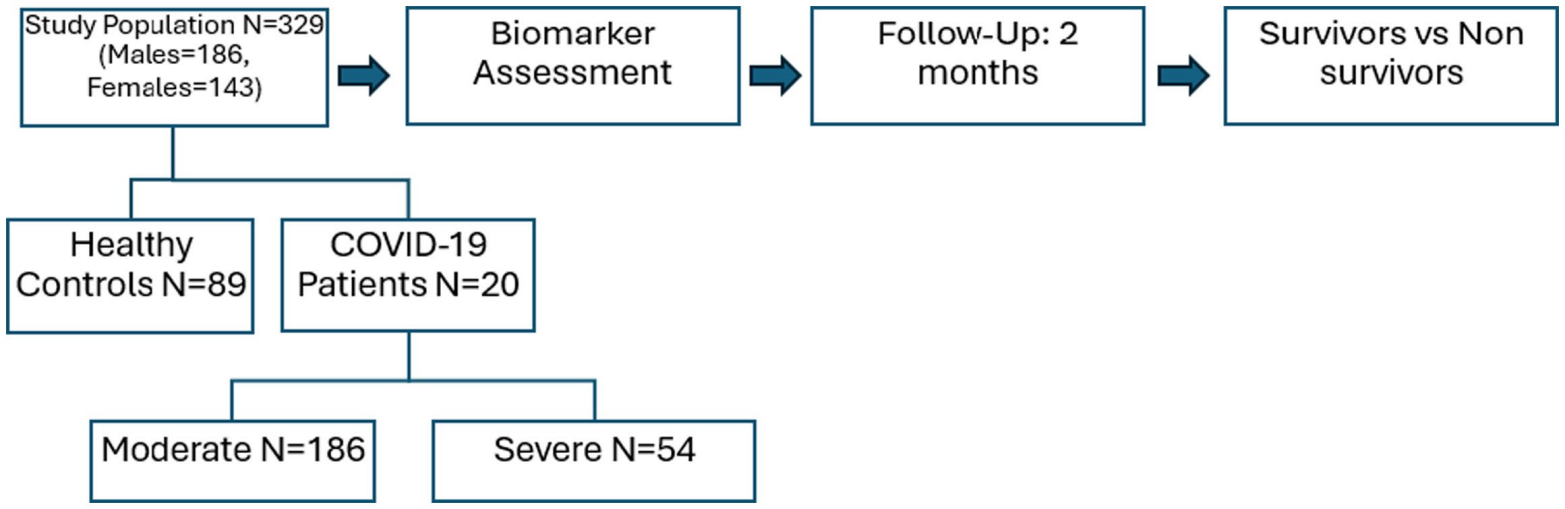
Schematic presentation of the study.

The ethical review committee of the King Edward Medical University, Lahore (No.304/RC/KEMU) endorsed the present prospective cohort study. For this purpose, Ittefaq Hospital and Mayo Hospital were visited to collect the blood samples of RT-PCR-positive COVID-19 patients during the 2021–2022. A questionnaire was designed for the demographic and clinical details of participants. Before the recruitment process, the study plan was explained to each participant or guardian and written informed consent was taken. Patients with a history of any infectious disease, diabetic patients, hypertension, or comorbidity with cardiovascular disease were excluded from this study. For this analysis, blood samples of COVID-19 patients (*n* = 240) were collected, out of which 186 were moderate and 54 were severe. The healthy subjects (*n* = 89) as a control group of our study were also recruited. After that patients were followed up for 2 months and found that 212 patients survived while 28 were non-survivors (dead) (Figure 1).

All personal protective equipment and hygienic measures have been opted for as the investigation involved COVID-19 pandemic patient sampling. Blood samples (5 cc) were collected and poured into the coagulant activator vial for 30 min at 25°C. Following that, the blood samples were centrifuged at 3000 rpm for serum separation. Afterwards, the serum samples were kept at −80°C till biochemical analysis. ELISA was employed for the analysis of serum decorin (CAT# I4833) and biglycan (CAT# I4351) levels by using commercially available ELISA kits of Glory Science (China).

**Figure 2:**
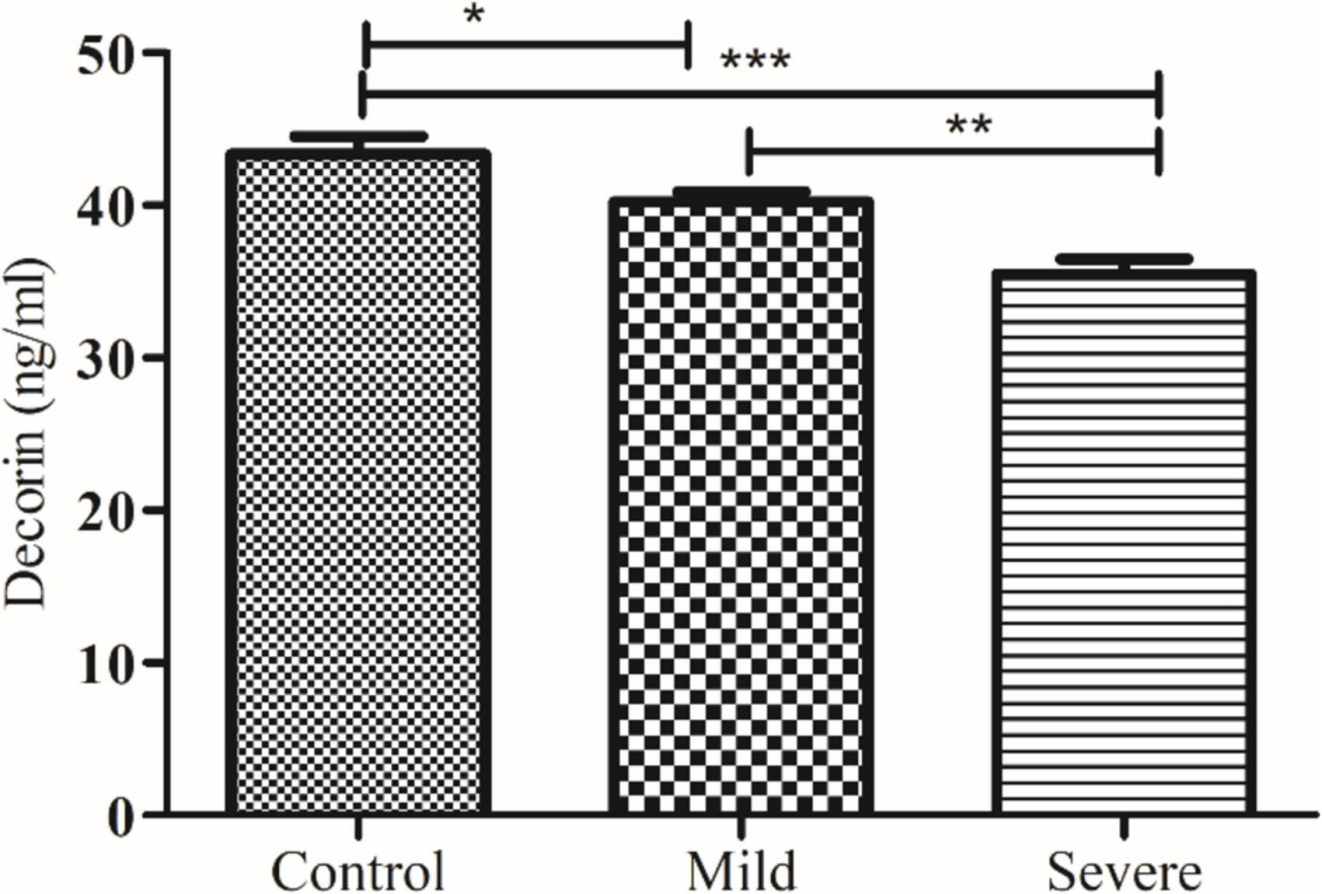
Comparison of serum decorin level in control and patients with moderate and severe COVID-19. *, ** and *** indicate significance at *p* < 0.05, 0.01 and 0.001, respectively.

**Figure 3:**
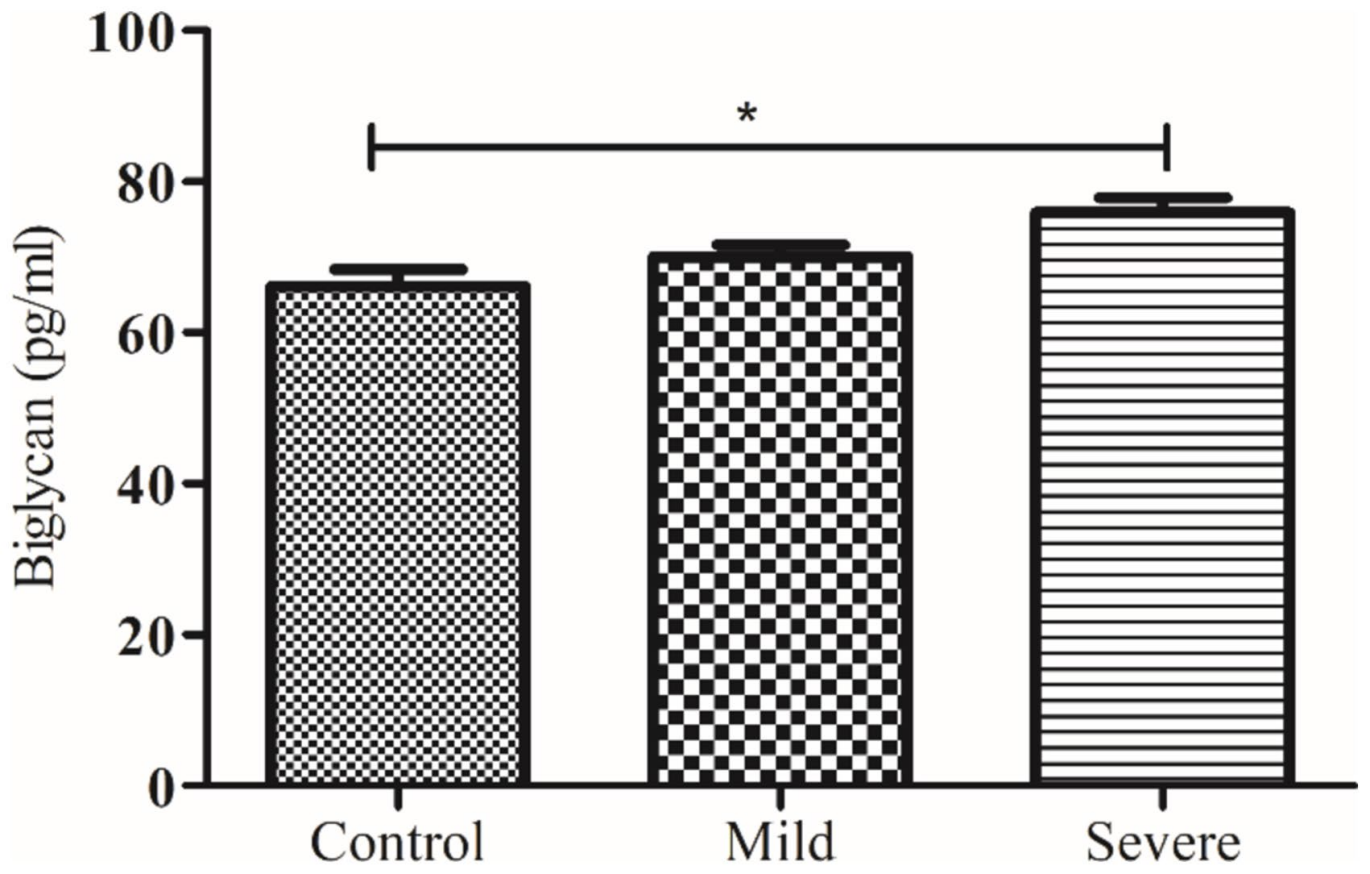
Comparison of serum biglycan level in control and patients with moderate and severe COVID-19. * indicate significance at *p* < 0.05.

**Figure 4:**
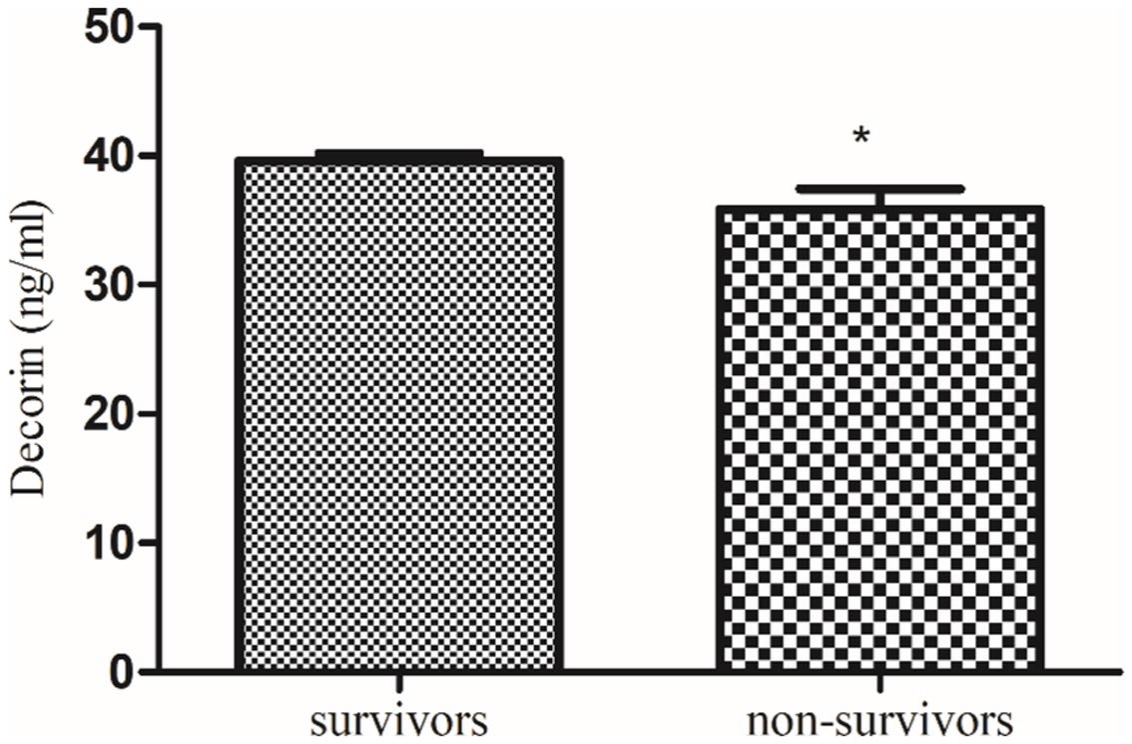
Comparison of serum decorin level in COVID-19 survivors and non-survivors. * indicate significance at *p* < 0.05.

**Figure 5:**
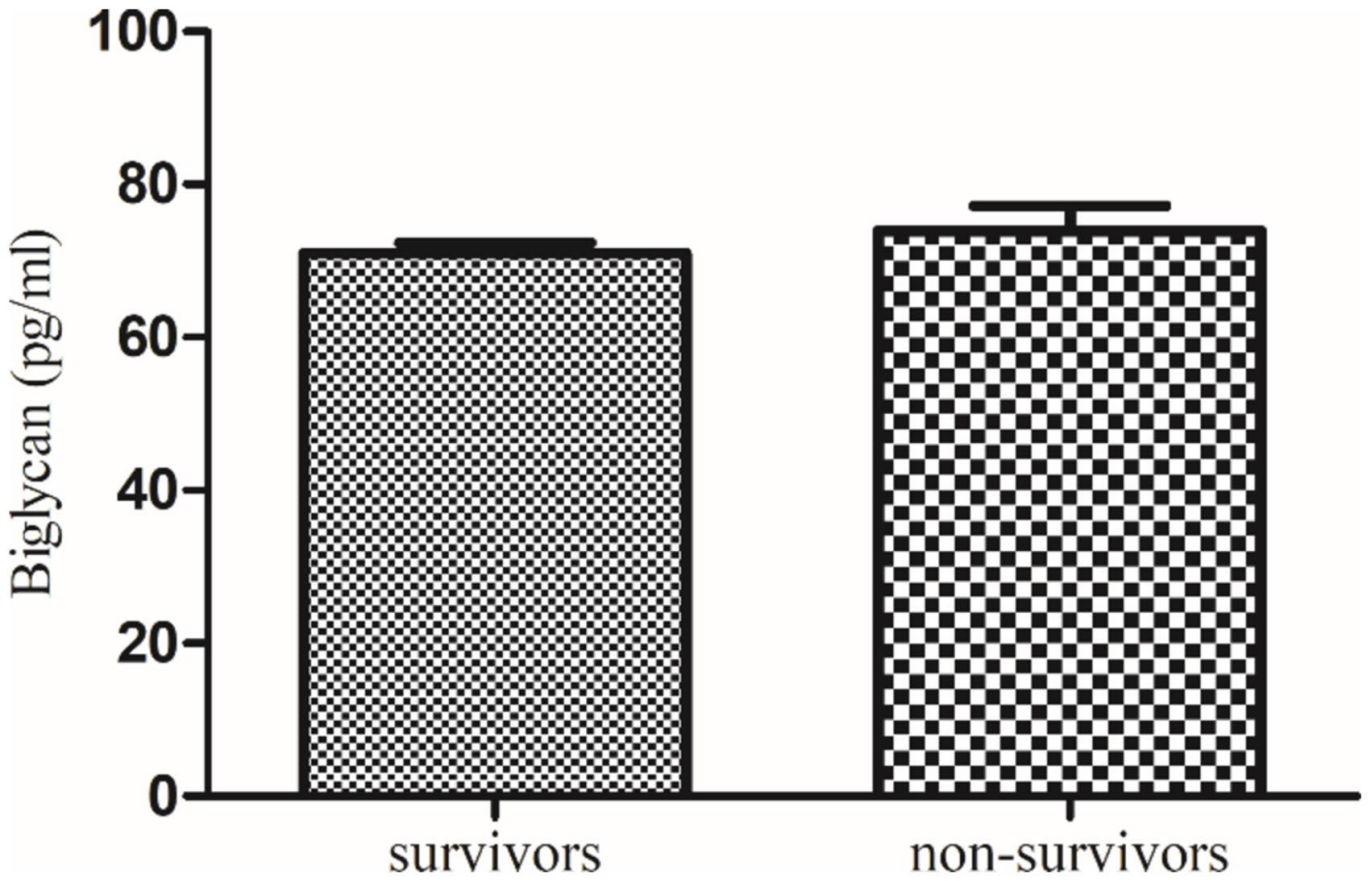
Comparison of serum biglycan level in COVID-19 survivors and non-survivors.

**Figure 6:**
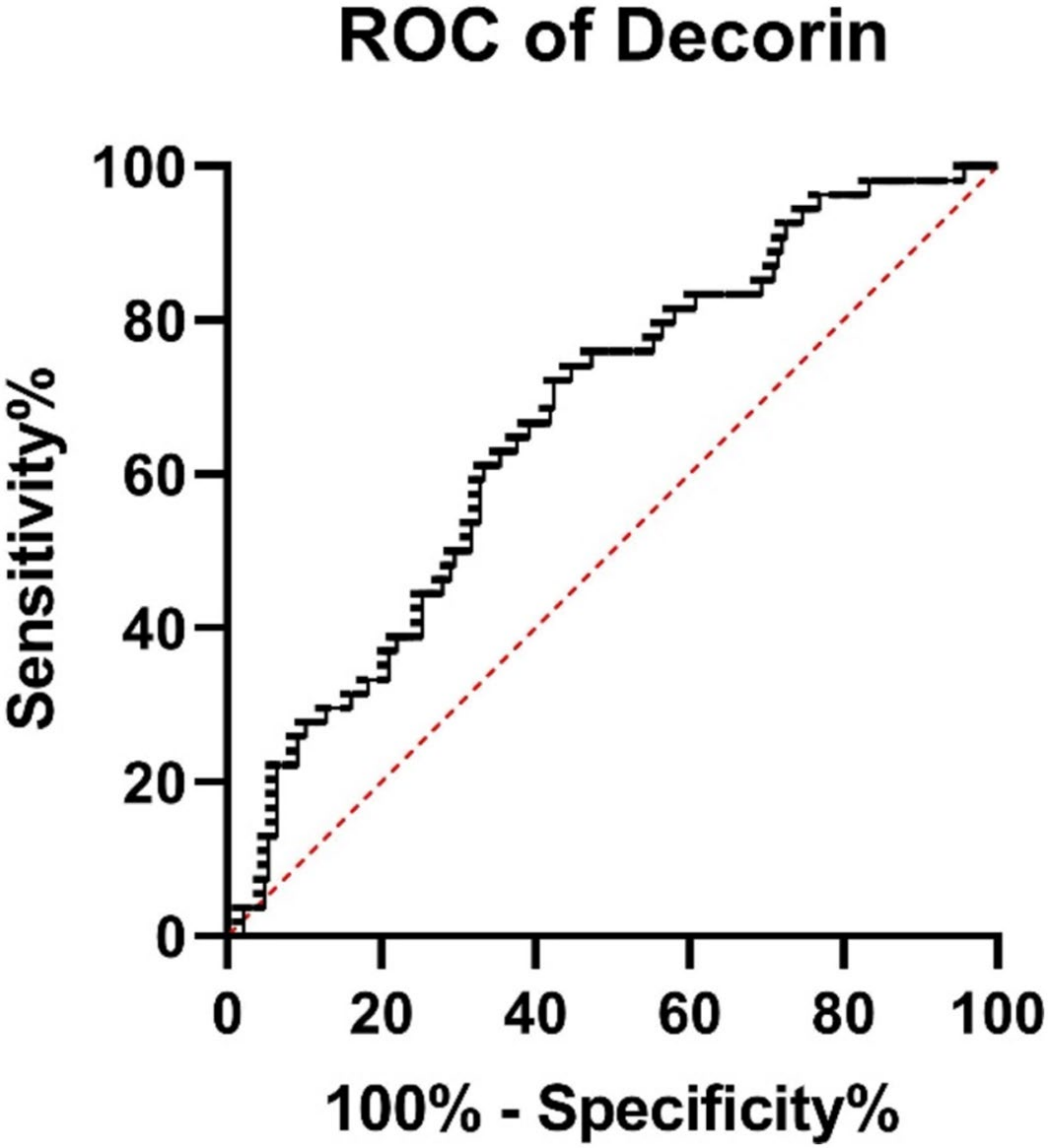
ROC curves for decorin illustrate the discriminatory power of each test.

**Figure 7:**
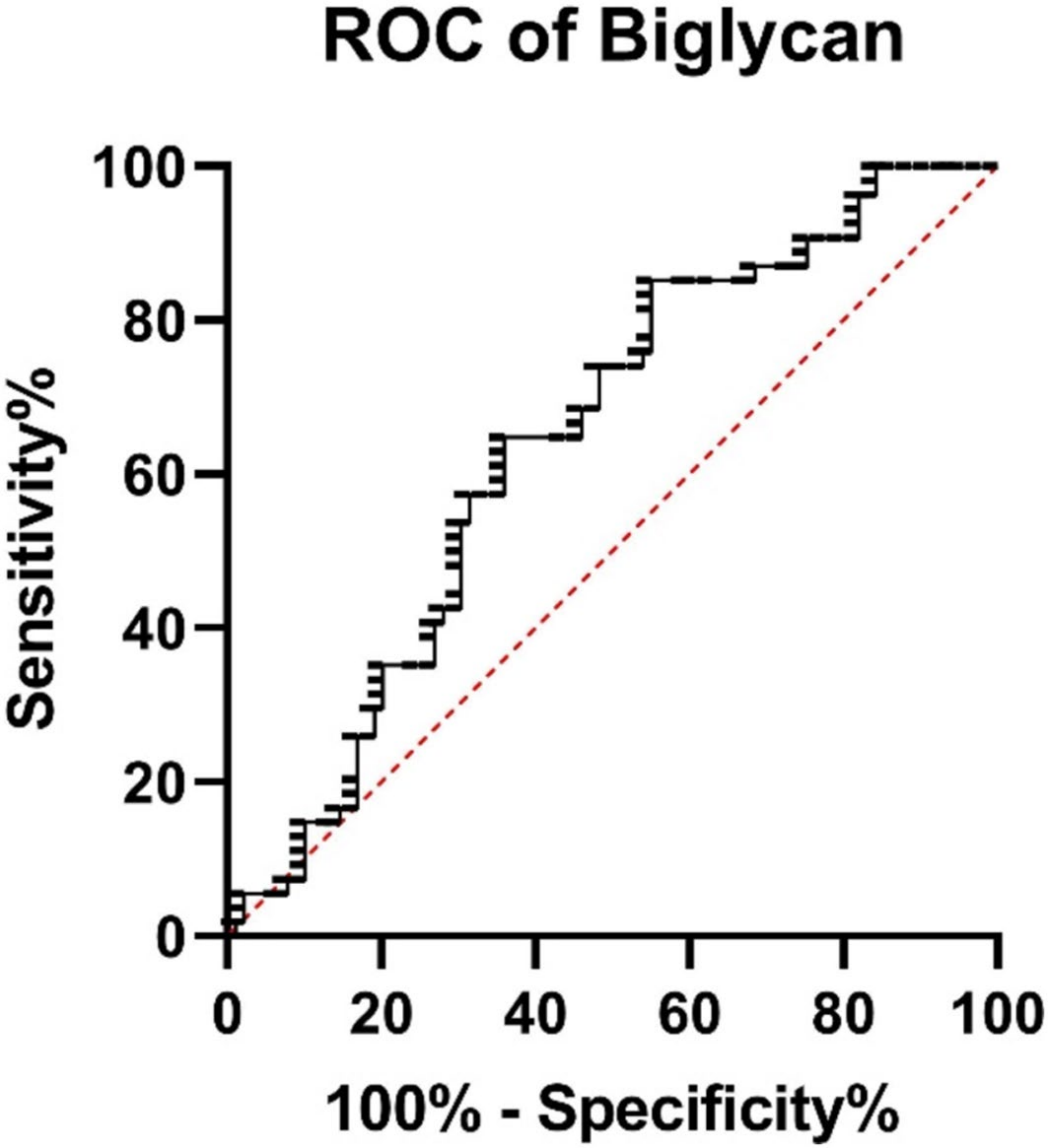
ROC curves for biglycan illustrate the discriminatory power of each test.

Obtained results were statistically analyzed by using GraphPad Prism (version 6.01), applying the student ‘*t*’ test and One-Way ANOVA with a significance level of *p* < 0.05, and the results were reported as mean ± SEM. Chi-Square Test for Association was also applied on demographic features. Receiver Operating Characteristic (ROC) analyses were also conducted to evaluate the discriminatory power of biomarkers (Figures 2–7).

## 3 Results

The demographic attributes of the participants at the time of sampling are described in Table 1. The Chi-Square analysis showed significant associations between COVID-19 severity and several parameters. Age groups (*p* = 0.014), BMI categories (*p* = 0.014), and residence type (*p* = 0.029) were all significantly linked to severity, with notable trends observed. Hospital stay duration was also significantly associated with severity (*p* = 0.002). Marital status and gender did not show significant links, while mortality was strongly related to severity (*p* = 0.000), emphasizing its impact on patient outcomes.

**Table 1:** Socio demographic factors of the study subjects.

Parameters	Category	Control (*N* = 89)		Mild (*N* = 186)		Severe (*N* = 54)		Chi-Square
		*N*	%	*N*	%	*N*	%	*p* value
Age (Years)	<18	12	13.48	17	9.14	3	5.56	0.014
	18–40	35	39.33	93	50.00	15	27.78	
	41–60	34	38.20	56	30.11	24	44.44	
	>60	8	8.99	20	10.75	12	22.22	
BMI (Kg/m^2^)	Underweight	13	14.61	17	9.14	2	3.70	0.014
	Normal	47	52.81	67	36.02	24	44.44	
	Overweight	19	21.35	72	38.71	17	31.48	
	Obese	10	11.24	30	16.13	11	20.37	
Education	Literate	62	69.66	123	66.13	37	68.52	0.848
	Illiterate	27	30.34	63	33.87	17	31.48	
Residence	Rural	53	59.55	139	74.73	40	74.07	0.029
	Urban	36	40.45	47	25.27	14	25.93	
Marital status	Unmarried	22	24.72	42	22.58	42	77.78	0.000
	Married	67	75.28	144	77.42	12	22.22	
Hospital stay (days)	0–5	NA	NA	61	32.80	5	9.26	0.002
	6–15,	NA	NA	90	48.39	32	59.26	
	>15	NA	NA	35	18.82	17	31.48	
Gender	Male	47	52.81	107	57.53	32	59.26	0.691
	Female	42	47.19	79	42.47	22	40.74	
Mortality	survivors	NA	NA	175	94.09	37	68.52	0.000
	non-survivors	NA	NA	11	5.91	17	31.48	

Among clinical symptoms fever and cough were most prevalent (69.15 and 62.77%, respectively) clinical symptoms among all the patients. While diarrhea, ICU admission and olfactory dysfunction were more prevalent (23.08, 30.77 and 50.00%, respectively) among the Severe patients.

The levels of decorin and biglycan were measured across three groups: controls, patients with moderate COVID-19, and patients with severe COVID-19, with results expressed as mean ± SEM. decorin levels in the control group were 43.36 ± 1.14 ng/mL, which significantly decreased in patients with moderate COVID-19 (40.24 ± 0.64 ng/mL) and further decreased in severe COVID-19 patients (35.49 ± 1.00 ng/mL), with an ANOVA *p* value of 0.0059. Conversely, biglycan levels showed an increasing trend, being 66.15 ± 2.22 pg/mL in the control group, 70.02 ± 1.57 pg/mL in moderate COVID-19 patients, and 75.88 ± 1.97 pg/mL in severe COVID-19 patients, with an ANOVA p value of 0.0042. These results indicate that while decorin levels decrease with the severity of COVID-19, biglycan levels increase, highlighting significant biochemical alterations associated with the disease’s progression.

Further, a follow-up study of these recruited qRT-PCR-positive COVID-19 patients revealed that serum decorin was significantly higher in survivors (39.6 ± 0.59 ng/mL) compared to non-survivors (35.84 ± 1.61 ng/mL) with a *p* value of 0.0319. On the other hand, serum biglycan levels were non-significantly lower in survivors (70.98 ± 1.41 pg/mL) compared to non-survivors (73.99 ± 3.24 pg/mL) with a p value of 0.459. These findings suggest a potential correlation between higher decorin levels and survival in COVID-19 patients, while changes in biglycan levels were not significantly associated with patient outcomes.

The ROC analysis revealed that *decorin* has an AUC of 0.665 (95% CI: 0.5868–0.7433, *p* = 0.0002), showing modest discriminatory power between survivors and non-survivors (Figure 6). *Biglycan* demonstrated an AUC of 0.639 (95% CI: 0.5481–0.7299, *p* = 0.0054), indicating acceptable differentiation between groups (Figure 7).

## 4 Discussion

Clinically confirmed lung fibrosis was observed in 56% of moderate COVID-19 and in 71% of patients with severe symptoms, 3 months after their recovery from Coronavirus. Lung fibrosis was clinically confirmed in 56% of the patients who experienced moderate COVID-19 symptoms and in 71% of the patients with severe symptoms, 3 months after they recovered (10).

Our study reveals a significant decrease in decorin levels in COVID-19 patients. This decrease is more pronounced in patients with severe COVID-19 compared to those with moderate symptoms. Additionally, follow-up data indicate that decorin levels were significantly lower in non-survivors compared to survivors. Previously, de Souza Xavier Costa, Ribeiro (11) reported decreased decorin in COVID-19 patients compared to non-COVID-19 patients, as demonstrated by the current study, severe COVID-19 patients exhibit the lowest levels of decorin compared to the control group.

These findings suggest that lower decorin levels may be associated with disease severity and poor prognosis in COVID-19 patients. Decorin regulates fibrillogenesis as an anti-fibrotic agent, partly by binding to and neutralizing TGF-*β* (12). TGF-β1 can reduce decorin synthesis in fibroblasts, while conversely, decorin can suppress TGF-β1 (13). It can also bind to collagen fibrils, assisting in the strengthening of collagen (14). A reduction in decorin levels can heighten the susceptibility to lung fibrosis. Additionally, decorin can suppress connective tissue growth factor-induced collagen formation in fibroblasts (15).

Another major finding of our study demonstrates that serum biglycan levels are significantly elevated in severe COVID-19 patients compared to healthy controls. However, follow-up data indicate that there is no significant difference in serum biglycan concentrations between survivors and non-survivors. This suggests that while biglycan levels are associated with disease severity, they may not be a reliable marker for predicting patient outcomes. Previously, in an investigation, it was found that the expression of the biglycan gene increases notably in COVID-19 cases after more than 7 days of ventilator management. Unlike decorin, biglycan potentially plays a role in sequestering TGF-*β* to cell-surface receptors, thereby promoting the formation of ECM (16).

Moreover, therapeutic replacement or enhancement of decorin has the potential to benefit as an immunomodulatory therapy, since it is known to modulate VEGF, TGFb and EGFR pathways (17).

Decorin and biglycan are crucial for integrity of the ECM and regulating cellular responses to growth factors and cytokines (18). In severe COVID-19 cases, abnormal ECM remodeling has been associated with pulmonary inflammation and fibrosis, suggesting a potential role for decorin and biglycan in these pathological processes (19). Granzyme B, an immune protease, can cleave decorin and biglycan, releasing active TGF-β1, which is involved in fibrosis and immune regulation (20). Additionally, elevated levels of inflammatory cytokines in COVID-19 patients may impact the expression and activity of these proteoglycans, further influencing their roles in disease progression (21).

Using aerosol delivery to administer decorin, particularly to the lungs, could offer enhanced efficacy against SARS-CoV-2. Moreover, a formulation based on decorin could be administered via a route that ensures high level of bioavailability. Decorin could enhance pulmonary function during SARS-COv-2-related problems, such as lung fibrosis. Furthermore, decorin can function as an adjunctive treatment when combined with other medicines, offering a reciprocal application in COVID-19 therapy (22).

This study has several limitations. The small, specific population from two hospitals in Lahore, Pakistan, limits the generalizability of the findings. The two-month follow-up period may not capture the long-term impact of COVID-19 on lung fibrosis. The study did not account for confounding factors like pre-existing conditions or medications. Lastly, variability in ELISA assays could affect biomarker measurement accuracy. Future studies should include larger, more diverse populations, longer follow-up periods, and additional biomarkers to validate these findings.

Conclusively, our has found that severe COVID-19 patients had significantly lower serum decorin levels and higher biglycan levels compared to healthy controls. These findings suggest that severe COVID-19 patients are at a higher risk of developing lung fibrosis and inflammation. Monitoring these biomarkers could help identify patients at risk for severe pulmonary complications, aiding in their management and treatment.

## Data Availability

The original contributions presented in the study are included in the article/supplementary material, further inquiries can be directed to the corresponding author.
